# A cross-sectional study of socio-demographic factors associated with patient access to primary care in Slovenia

**DOI:** 10.1186/s12939-015-0166-y

**Published:** 2015-04-21

**Authors:** Suzana Kert, Igor Švab, Maja Sever, Irena Makivić, Danica Rotar Pavlič

**Affiliations:** Department of Family Medicine, Ljubljana Medical School, Poljanski nasip 58, 1000 Ljubljana, Slovenia; Department of Family Medicine, Maribor Medical School, Maribor, Slovenia; Statistical Office of the Republic of Slovenia, Ljubljana, Slovenia

**Keywords:** Economic recession, Access to primary care, Slovenia, Patient perceptions, Socio-demographic factors

## Abstract

**Introduction:**

Primary care (PC) is the provision of universally accessible, integrated, person-centred, comprehensive health and community services. Professionals active in primary care teams include family physicians and general practitioners (FP/GPs). There is concern in Slovenia that the current economic crisis might change the nature of PC services. Access, one of the most basic requirements of general practice, is universal in Slovenia, which is one of the smallest European countries; under national law, compulsory health insurance is mandatory for its citizens. Our study examined access to PC in Slovenia during a time of economic crisis as experienced and perceived by patients between 2011 and 2012, and investigated socio-demographic factors affecting access to PC in Slovenia.

**Methods:**

Data were collected as a part of a larger international study entitled Quality and Costs of Primary Care in Europe (QUALICOPC) that took place during a period of eight months in 2011 and 2012. 219 general practices were included; in each, the aim was to evaluate 10 patients. Dependent variables covered five aspects of access to PC: communicational, cultural, financial, geographical and organizational. 15 socio-demographic factors were investigated as independent variables. Descriptive statistics, factor analysis and multilevel analysis were applied.

**Results:**

There were 1,962 patients in the final sample, with a response rate of 89.6%. The factors with the most positive effect on access to PC were financial and cultural; the most negative effects were caused by organizational problems. Financial difficulties were not a significant socio-demographic factor. Greater frequency of visits improves patients’ perception of communicational and cultural access. Deteriorating health conditions are expected to lower perceived geographical access. Patients born outside Slovenia perceived better organizational access than patients born in Slovenia.

**Conclusions:**

Universal medical insurance in Slovenia protects most patients from PC inaccessibility. However, problems perceived by patients may indicate the need for changes in the organization of PC.

**Electronic supplementary material:**

The online version of this article (doi:10.1186/s12939-015-0166-y) contains supplementary material, which is available to authorized users.

## Introduction

### Trends in society

The world is currently facing the most severe financial and economic crisis in decades, affecting families and communities [1], and many European governments have decided to cut back on public expenditure on health [2]. Several countries, including Slovenia, have introduced measures that include increasing taxes on alcohol and tobacco, abandonment or scaling down of planned investments, reduction in the number of health sector workers and their pay, shifting the preventive activities of GPs to registered nurses, adoption of strategies to lower waiting times, improvements in prescribing drugs, and improving the use of health assessment technology [3].

### The importance of primary (health) care

Many authors consider universally accessible PC to be a cornerstone of strong health-care systems [4,5]. The Expert Panel of the European Commission has recently formulated an up-to-date concept of primary (health) care. Under this definition, universally accessible, integrated, person-centred, comprehensive health and community services must be provided by a team of professionals able to address most personal health needs. These services are to be delivered in a sustained partnership between patients and formal caregivers, in the context of family and community, and play a central role in the overall coordination and continuity of people’s health care needs. Reflecting widespread usage, we use the terms “primary care” and “primary health care” interchangeably. Accessibility of health services affects different aspects of the structure of health services and health facilities, including access to health care practitioners in terms of location, time, and ease of approach [6]. Dutch researchers defined access to PC even more exhaustively in terms of seven features: availability, geographic accessibility, accommodation of accessibility, affordability, acceptability, utilization, and equity in access [7]. Equality in health care is one of the crucial features that quality PC should have [8]; the outcome of a PC system includes three dimensions: quality of care; efficiency of care; and equity in health [7].

### Economic crisis and PC in Slovenia

Slovenia is a Central European country with 2.1 million inhabitants. In 1991, Slovenia became independent [9] and a year later its health care system was transformed from a state-run system to a decentralized model with one insurance company [10]. The Slovenian economy was growing rapidly when the country joined the euro zone in 2007, but this growth surge was fueled by debt. The budget deficit rose significantly, and restoring public finances has proved difficult [11]. Total health-care spending in Slovenia in 2012 accounted for 9.4% of gross domestic product, close to the 9.3% average in OECD countries. Health spending in Slovenia decreased markedly following the economic crisis and was negative in real terms in 2010. Since then, expenditure on health has started to grow again in 2011 and 2012, but at a very modest rate (under 1% in real terms per year) [12].

As a result of the financial crisis, the unemployment rate in Slovenia rose from 7% in 2008 [13] to 12% in 2012 [14]. In the same year, the elderly population (age 65 and over) accounted for 16.9% of the total population [15]. In interviews with a number of adults in 2011, 60.1% estimated their health as good or very good [16], 36% reported a long-standing illness or health problem [17], and 66.3% reported at least one moderate problem [18].

Health care in Slovenia is funded by a mix of public and private spending. The public sector is the primary source of health care funding. On average across EU countries, three-quarters of all health care spending was publicly funded in 2012 [19]. Slovenia’s health system is funded by compulsory health insurance for everyone meeting statutory requirements [20], by state revenues, voluntary health insurance, and out-of-pocket spending.

The delivery of PC is organised in health care centres and health stations. Health care personnel involved in PC include FP/GPs [9]. FPs in Slovenia act as “gatekeepers,” controlling access to secondary services. Patients must choose their own personal FPs, who are responsible for providing PC for their patients, including emergency care 24 hours a day provided by physicians working in rotation outside regular office hours [10]. This requirement has had a great impact on both the quality and cost of health care [21,22].

Most first-patient contacts are made by FPs, and continued good access is of the utmost importance. Low or unequal access results in low patient satisfaction [23,24]. Previous studies have examined several factors affecting access: having a relationship with a PC source with characteristics of a medical center [24,25], the availability of timely [25-28] and/or easy phone access [25,27], after-hours care, physician knowledge of the patient’s medical history, adequate time allotted to consultation [25], the attitude on the phone of the doctor’s assistant, patient opinion of FP treatment, waiting time [26], the ability to obtain an outpatient appointment for the same or following day [24], time spent in the waiting room, and seeing the same FP most of the time [27].

Studies have already shown many patient characteristics that adversely affect access. These include: old age [24], economic factors [24,29,30], chronic health problems [24] and membership in ethnic groups [28,31]. Lower access results in fewer patients visits [32,33].

Many international authors are of the opinion that more research is needed to assess the impact of policies that support free and universal access to public services, and about individuals’ experience and perceptions of the effects of the financial crisis on health care [34].

Our study aimed to determine how Slovenian patients experience and perceive different aspects of access to PC in a time of economic crisis and austerity measures, and to identify potential socioeconomic factors that could influence perceived (good or bad) access to PC.

## Material and methods

### Data source

Between September 2011 and April 2012, we performed a cross-sectional observational study of Slovenian general practices. The study was conducted as part of an international study known as Quality and Costs of Primary Care in Europe (QUALICOPC). The detailed protocol for this study has already been published elsewhere [35,36].

Based on this study protocol, patients were approached through their treating physician. 219 Slovenian general practices were included in the study, with each aiming to survey ten consecutive patients. Nine patients filled in the standard questionnaire about the experiences and one patient about what he or she found important when visiting a general practice. In this study, the socio-demographic characteristics of study respondents were compared to those of the general population. This comparison showed that respondents were more female, less unemployed [37] and had more chronic health conditions than general population [19].

Our study explored patients’ experiences and perceptions of access to PC in Slovenia. Targeted sample size was 2,190 patients (219 practices aimed to survey 10 consecutive patients). Response rate was 89.6%, thus the final sample included 1,962 Slovenian patients. Response rate was calculated as number of patients in final sample size over targeted sample size (1,962/2,190 = 0.896*100 = 89.6%). The research protocol was approved on August 11, 2011 by the Commission of the Republic of Slovenia for Medical Ethics (decision number 144/07/11).

### Variables of interest

In order to investigate access to PC, we created variables covering communicational, cultural, financial, geographical and organizational aspects. Access to PC was defined as the dependent variable. We constructed indicators to measure levels of PC access covering five dimensions. For each aspect (dimension), we selected an initial set of conceptually relevant but mutually exclusive items: six items for communication, four for culture and organization, and two for financial and geographical factors. Items included in the concept of access to PC are detailed in Additional file 1: Annex 1.

Before summarizing item values referring to certain aspects of access, the underlying structure of selected items was explored and empirically tested using factor analysis (FA). For each dimension, we tested whether the obtained set of data really measured the same construct of interest (latent dimension) and checked its factor loadings. The results of the FA are summarized in Table 1. Five dependent variables on access to PC were deduced as the sum of item values; the highest value on the summarized scale was labelled as good access and 0 as bad access. In place of independent variables, we selected 15 socio-demographic characteristics of patients, including sex, age, place of birth, mother’s place of birth, language proficiency in Slovene, educational attainment level, activity status in terms of being unemployed, student or retired, material status considering household income, household composition as regards adults and children, self-assessed general health, presence of longstanding (chronic) health conditions such as high blood pressure, diabetes, depression, asthma or other longstanding conditions, and number of visits and consultations patient made in the previous six months. All dependent and independent variables used in the analysis are presented in Table 2.

**Table 1:** **Factor analysis of manifest questions for access to PC dimensions**

**Access to PC dimension**	**Manifest questions**	**Initial eigenvalue**	**Variance explained**	**Total variance explained**	**Factor solution**	**Factor loading**
Communicational	medirec, polite, listen, questhl, timesuf, involve	2.278	38.0	38.0	1	0.419
		0.898	15.0	52.9		0.691
		0.860	14.3	67.3		0.717
		0.742	12.4	79.6		0.607
		0.682	11.4	91.0		0.660
		0.540	9.0	100.0		0.554
Cultural	disrethn, disrsex, actneg, bettertr	2.094	52.4	52.4	1	0.769
		0.919	23.0	75.3		0.764
		0.564	14.1	89.4		0.701
		0.423	10.6	100.0		0.653
Financial	postpinsur, postpfinan	1.164	58.2	58.2	1	0.763
		0.836	41.8	100.0		0.763
Geographical	farpract, travel	1.404	70.2	70.2	1	0.838
		0.596	29.8	100.0		0.838
Organizational	homevis, easyapp, waitdays, diffoohr	1.457	36.4	36.4	1	0.647
		0.943	23.6	60.0		0.598
		0.816	20.4	80.4		0.576
		0.783	19.6	100.0		0.591

**Table 2:** **Independent and dependent variables used in the analysis**

**Variables**	**Values**
Independent variables	
Sex	1 = male, 2 = female
Age	years
Place of birth	1 = Slovenia, 2 = another country
Mother’s place of birth	1 = Slovenia, 2 = another country
Language proficiency	1 = fluent/native speaker, 2 = sufficiently, 3 = moderately, 4 = poorly, 5 = not at all
Educational attainment level	1 = primary or less, 2 = upper secondary, 3 = post-secondary or higher
Unemployed	1 = yes, 0 = no
Student	1 = yes, 0 = no
Retired	1 = yes, 0 = no
Household income	1 = below average, 2 = average, 3 = above average
Household with other adults^1^	1 = yes, 0 = no
Household with children^2^	1 = yes, 0 = no
Health status	1 = very good, 2 = good, 3 = fair, 4 = poor
Longstanding condition	1 = yes, 0 = no
Number of visits/consultations^3^	1 = one, 2 = two, 3 = three to five, 4 = six or more
Dependent variables	
Communicational access	0 to 6 (0 = bad access, 6 = good access)
Cultural access	0 to 4 (0 = bad access, 4 = good access)
Financial access	0 to 2 (0 = bad access, 2 = good access)
Geographical access	0 to 2 (0 = bad access, 2 = good access)
Organizational access	0 to 4 (0 = bad access, 4 = good access)

Notes: ^1^including children older than 18; ^2^under 18, ^3^considering last 6 months.

### Statistical analysis

Descriptive statistics were used to analyze key features of the patient sample, considering socio-demographic characteristics and perceived access to PC. Since individual patients were nested within general practices, we performed a multilevel analysis to partition the variance in access to PC aspects attributable to individual patient level and to higher general practice level. Interclass correlation (ICC) was calculated to assess the proportion of variance at the patient level. To identify the socio-demographic factors affecting access to PC, multilevel regression models were estimated using access to PC dimensions as dependent variables and socio-demographic factors as predictors. The effects of socio-demographic characteristics on perceived access to PC were investigated using unstandardised regression coefficients. To interpret them properly, their scales must be taken into account. Our main goal was to consider the socio-demographic factors with a statistically significant effect on access to PC. The overall fit of the estimated multilevel models was tested using chi-square (χ^2^) likelihood-ration (LR) test, i.e. deviance, which is defined as minus twice the log-likelihood (−2LL). This was used to compare the performance of the null model (without any covariates) and level 1 model (including 15 patient level covariates). The confidence level was set at p < 0.05. Analyses were performed using IBM SPSS Statistics for Windows (version 22.0).

## Results

Table 3 shows the results of the descriptive statistics of the patient sample considering socio-demographic characteristics. The final sample consisted of 1,962 patients (89.6% response rate). 59.1% of patients were female. The average patient age was 48.7±16.9 years. Most patients and their mothers were born in Slovenia. 11.5% of patients were born outside Slovenia, while 17.2% reported that their mother’s country of birth was a country other than Slovenia. Most (92.7%) patients declared themselves to be fluent or native speakers of Slovene. 44.5% of patients surveyed had upper secondary level education, followed by primary level or lower (31.2%), and post-secondary level or higher (24.3%). 6.6% of patients were unemployed, 6.5% were students and 30.5% were retirees. More than half of the patients stated that their household income was average (58.5%), while 32.5% identified below average household income and 9.0% reported living in a household with above average income. 79.7% of patients lived in households with other adults (including children above 18) and 34.0% in households with children under 18. 12.5% of patients self-assessed their general health status as very good and 12.3% as poor. Most, however, judged themselves to be in good health (41.2%). 45.9% of patients confirmed having at least one longstanding condition. In terms of number of visits and consultation in the previous six months, 31.8% of patients had made three to five visits.

**Table 3:** **Socio-demographic characteristics of patient sample (N=1,962)**

**Variables**		**Number of patients**
Sex	male	793 (40.9%)
	female*	1,146 (59.1%)
Age (in years)	mean (sd)	48.7 (16.9)
Place of birth	Slovenia*	1,716 (88.5%)
	another country	222 (11.5%)
Mother’s place of birth	Slovenia*	1,609 (82.7%)
	another country	334 (17.2%)
Language proficiency	fluent/native speaker*	1,791 (92.7%)
	sufficiently	100 (5.2%)
	moderately	34 (1.8%)
	poorly	6 (0.3%)
	not at all	2 (0.1%)
Educational attainment level	primary or less	602 (31.2%)
	upper secondary*	859 (44.5%)
	post-secondary or higher	469 (24.3%)
Unemployed	yes	128 (6.6%)
	no*	1,807 (93.4%)
Student	yes	126 (6.5%)
	no*	1,809 (93.5%)
Retired	yes	590 (30.5%)
	no*	1,345 (69.5%)
Household income	below average	628 (32.5%)
	around average*	1,132 (58.5%)
	above average	174 (9.0%)
Household with other adults	yes*	1,540 (79.7%)
	no	393 (20.3%)
Household with children	yes	659 (34.0%)
	no*	1,278 (66.0%)
Health status	very good	246 (12.5%)
	good*	807 (41.2%)
	fair	666 (34.0%)
	poor	242 (12.3%)
Longstanding condition	yes	901 (46.0%)
	no*	1,057 (54.0%)
Number of visits/consultations	one	509 (26.7%)
	two	549 (28.8%)
	three to five*	607 (31.8%)
	six or more	244 (12.8%)

Notes: * prevailing category.

For each dimension of access to PC, a single latent variable was created. Key points associated with each dimension are:

- communicational dimension relates to communicational features of consultation (using medical records, politeness, active listening, sufficient time mode, acts in partnership);
- cultural dimension comprehends the relationship of the physician and his or her staff to the patient in terms of respectful behavior, ethnic and gender equality;
- financial dimension includes financial reasons and insurance problems resulting in postponement of visiting the physician;
- geographical dimension refers to access in terms of distance and travel time from patients’ homes and workplaces to the physicians’ offices.
- organizational dimension relates to organizational aspects of the physicians’ offices (waiting time, uncomplicated appointment mode, office hours, home visit options).

Descriptive statistics of access to PC as experienced and perceived by patients are given in Table 4. All five dimensions recorded a high concentration of patients in the upper part of the scale, indicating good access to PC. Very little variation was found for cultural and financial access to PC; more than 95% of Slovenian patients scored the highest possible level of access. 95.3% of patients reported that they had not experienced any disrespectful behavior regarding their ethnic background or gender, or negative or uncaring attitude by a doctor or other staff member, and had never had the feeling that other patients received better treatment. On the other hand, three (0.2%) patients reported experiencing all the problems listed. According to patient experience data, financial access to PC proved to be good. 99.3% of Slovenian patients had no financial problems that substantially limited their access to PC, while 0.7% of patients confirmed having insurance or financial problems resulting in postponing or cancelling a visit.

**Table 4:** **Access to PC experienced and perceived by patients**

**Access to PC dimension**		**Number of patients (share)**	**Mean(sd)**
Communicational (N=1,943)	0 = bad access	3 (0.2%)	5.548 (0.888)
	1	18 (0.9%)	
	2	17 (0.9%)	
	3	29 (1.5%)	
	4	111 (5.7%)	
	5	393 (20.2%)	
	6 = good access*	1,372 (70.6%)	
Cultural (N=1,593)	0 = bad access	3 (0.2%)	3.930 (0.356)
	1	4 (0.3%)	
	2	19 (1.2%)	
	3	49 (3.1%)	
	4 = good access*	1,518 (95.3%)	
Financial (N=1,928)	0 = bad access	1 (0.1%)	1.992 (0.094)
	1	13 (0.7%)	
	2 = good access*	1,914 (99.3%)	
Geographical (N=1,874)	0 = bad access	50 (2.7%)	1.860 (0.417)
	1	163 (8.7%)	
	2 = good access*	1,661 (88.6%)	
Organizational (N=1,292)	0 = bad access	5 (0.4%)	3.543 (0.732)
	1	21 (1.6%)	
	2	93 (7.2%)	
	3	322 (24.9%)	
	4 = good access*	851 (65.9%)	

Notes: *prevailing category.

Slightly more variation was observed in the communicational, geographical and organizational dimensions. 72.5% of Slovenian patients gave geographical access to PC the highest score. On the other hand, 2.7% found PC too far away from their home or workplace and reported spending more than 40 minutes travelling to PC. 70.6% of patients did not have any observed communicational difficulties in accessing PC. Only three (0.2%) patients reported some shortcomings related to their doctor’s medical records usage, politeness, paying attention, time mode and co-decision about further treatment. Organizational access showed the most variation of all five dimensions: 65.8% of patients in Slovenia reported no organizational problems when accessing PC, but in contrast, five (0.4%) patients assumed not getting a home visit if needed, perceived getting an appointment a difficult task, waited for more than a week for a visit, or found visiting a doctor during evening, nights and weekends to be challenging. Despite the fact that most patient responses were concentrated at the top of the accessibility scale, we still checked to see how much the variance resulted from differences at the patient level and how much at the higher general practice level. The results of partitioning variance are presented in Table 5. ICC values ranged from 85.8% to 96.5% and suggested that variation in patients’ experiences of access to PC stems predominantly from individual patient differences. Across all five dimensions, cultural and organizational factors showed the lowest ICCs. 14.2% of variance in cultural access proved to originate from differences between practices, but these were still outweighed by patient level differences. Considering that most variance arises from differences at the patient level, conditional models with 15 selected socio-demographic patient level covariates were estimated. The results are detailed in Table 6.

**Table 5:** **Multilevel models partitioning variance in access to PC to individual patient level and practice level**

**Access to PC dimension**	**Constant**	**Patient level variance**	**Practice level variance**	**ICC (%)**
Communicational	5.548	0.748	0.040	94.9
Cultural	3.928	0.109	0.018	85.8
Financial	1.992	0.008	0.000	96.2
Geographical	1.859	0.168	0.006	96.5
Organizational	3.534	0.470	0.070	87.1

Notes: ICC value is calculated as a proportion of individual patient level variance in total observed variance using all available decimal places, then presented in % to one decimal place.

**Table 6:** **Multilevel models of access to PC dimensions with individual patient level covariates**

**Fixed effects**	**Communicational access**	**Cultural access**	**Financial access**	**Geographical access**	**Organizational access**
**Coefficient (p-value)**					
Intercept	5.450 (0.000)**	3.787 (0.000)**	1.983 (0.000)**	2.040 (0.000)**	3.360 (0.000)**
Sex	0.047 (0.241)	-0.009 (0.603)	0.002 (0.665)	-0.056 (0.006)**	0.022 (0.586)
Age	0.000 (0.926)	0.002 (0.006)**	0.000 (0.092)	0.000 (0.960)	0.002 (0.285)
Place of birth	-0.109 (0.235)	-0.019 (0.617)	0.004 (0.686)	-0.028 (0.549)	0.197 (0.043)*
Mother’s place of birth	0.093 (0.210)	0.016 (0.618)	0.013 (0.140)	0.038 (0.323)	-0.073 (0.363)
Language proficiency	-0.053 (0.345)	-0.013 (0.583)	-0.003 (0.649)	-0.018 (0.531)	0.049 (0.448)
Educational attainment level	0.029 (0.324)	0.000 (0.982)	0.000 (0.965)	-0.012 (0.411)	-0.027 (0.370)
Unemployed	0.032 (0.700)	-0.016 (0.653)	-0.014 (0.137)	-0.033 (0.441)	-0.038 (0.663)
Student	-0.012 (0.893)	0.048 (0.206)	-0.019 (0.066)	-0.013 (0.773)	0.137 (0.148)
Retired	0.025 (0.709)	-0.019 (0.502)	0.012 (0.119)	-0.071 (0.041)*	-0.085 (0.220)
Household income	-0.037 (0.302)	0.015 (0.342)	0.003 (0.544)	0.026 (0.157)	-0.006 (0.875)
Household with other adults	0.052 (0.296)	0.034 (0.100)	0.008 (0.173)	0.016 (0.540)	0.078 (0.129)
Household with children	0.023 (0.606)	0.013 (0.479)	0.000 (0.974)	0.011 (0.635)	0.037 (0.421)
Health status	-0.031 (0.247)	-0.020 (0.074)	0.000 (0.935)	-0.043 (0.002)**	-0.049 (0.078)
Longstanding condition	-0.027 (0.554)	0.008 (0.681)	0.001 (0.857)	0.021 (0.362)	-0.009 (0.843)
Number of visits/consultations	0.071 (0.001)***	0.023 (0.010)**	0.001 (0.675)	0.003 (0.756)	0.007 (0.760)
Deviance	4,464.281	797.755	-3.371.262	1,903.012	45,001.437
χ^2^ change _(df change=15)_^1^	-570.775***	-371.134***	301.379***	-130.617***	-6,202.403***

Notes: *significance at the 0.05 level, **significance at the 0.01 level, ***significance at the 0.001 level, ^1^ Critical values for the χ^2^ statistics with 15 degrees of freedom are 24.996 (p<0.05), 30.578 (p<0.01), 37.697 (p<0.001).

The communicational access model showed only one significant regression coefficient, the number of visits and consultations each patient made in the previous six months. The regression coefficient is 0.071, which means that with each additional visit or consultation, the perceived communicational access is expected to increase by 0.071 scale points. It is also noteworthy that coefficients of place of birth, language proficiency, student status, household income, health status and longstanding conditions have a negative sign. This means that being born outside Slovenia, having poorer language proficiency, being a student, having higher household income, deteriorating health status and/or presence of a longstanding condition leads to lower communicational access to PC, but not significantly. Moreover, cultural access proved to significantly depend on only two socio-demographic factors, the patient’s age and the number of visits in the previous six months. The regression coefficient of age is 0.002. With each additional year of age, perceived cultural access is expected to increase by 0.002 scale points. In our study, age ranges from 17 to 93, so the predicted difference between the youngest and the oldest patient amounts to 0.152 scale points. The number of visits made in the previous six months proved to have a similar effect on cultural access as on communicational access; with each additional visit or consultation, the perceived cultural access is expected to increase by 0.023 scale points. The financial access model showed, surprisingly, no significant regression coefficient. However, three socio-demographic factors were found to have a negative sign. Poorer language proficiency, being unemployed or being a student leads to lower financial access, but not significantly. Furthermore, the geographical access model was found to have three significant regression coefficients, of sex, retired status, and health status. The coefficient of sex is −0.056, indicating that on average, female patients scored 0.056 lower on the geographical access measure compared to male patients. The coefficient of retired status is −0.071, and of health status, −0.043. From this we conclude that, on average, retirees scored 0.071 lower on perceived geographical access than non-retirees and that with each scale point of deteriorating health conditions, a patient perceived 0.043 points lower geographical access. In addition, another five coefficients have negative signs – being born outside Slovenia, poorer language proficiency, higher educational attainment level, being unemployed and being a student point to lower perceived geographical access, but not significantly. The organizational access model resulted in only one significant regression coefficient, place of birth. The regression coefficient is 0.197; this means that, on average, a patient born outside Slovenia scored 0.197 scale points higher on the organizational access scale compared to a patient born in Slovenia. Seven other regression coefficients have negative signs. Having a mother born outside Slovenia, higher educational attainment level, being unemployed, being retired, having higher household income, deteriorating health status and/or the presence of a longstanding condition all decrease perceived organizational access, but still not significantly.

The overall fit of the multilevel model with level 1 covariates was compared to a null model, examining change of deviance resulting from adding 15 socio-demographic covariates to a null model. All level 1 models, except the financial access model, showed improvement of the overall fit of a model measured by deviance. The change was also confirmed to be statistically significant (p < 0.05) for all four models.

## Discussion

In general, from patients’ reported experiences and perceptions, we can conclude that access to PC in Slovenia is very good. Unfortunately, no similar survey has been attempted before, so the data cannot be compared with experiences of patients during better economic conditions. One explanation for the result of this study is that most patients get access to PC in government-run health centres [38]. These health centres can be compared to medical centres in more economically developed countries than Slovenia, which have proven to be a very good source of health care [24,25]. Slovenian health centres have a long tradition and should maintain its mission and the basic principles, and would be upgraded with the new contents brought about by modern society [39].

In this study, the socio-demographic characteristics of study respondents were compared to those of the general population. This comparison showed that more respondents had chronic health conditions than the general population [19], which is to be expected, because in general practice there is a prevalence of sick persons, compared to the general population.

The best results regarding perceived access were observed in the communicational and financial aspects. In the communicational aspect of consultation, a very high proportion of patients had not experienced any inappropriate behavior or attitude by a doctor or other staff. Physician-patient communication, and the doctor’s knowledge of the patient’s medical history, has previously been confirmed as very important [20,25]. The financial aspect of patients’ experience with access to PC also turned out to be good, and this result did not come as a surprise. Good access to PC, in terms of communications and finances, was likewise consistent with previous studies [40]. In general, as shown in several previously published studies, patient satisfaction in Slovenia is among the highest in Europe [41-43]. The biggest variation was observed in organizational access, where about one third of patients reported various problems, and we assumed that this is related to financial cuts in health care in recent years [3].

We also checked how much variance results from differences at the patient level and how much at the general practice level. Our finding was that most variance originates at the patient level, and at this level, cultural and organizational aspects showed the lowest variance.

When we looked at different access models, the communicational and cultural access models showed as significant the number of visits and consultations patients made in the previous six months. It is known from previous research that a satisfactory level of continuity is a result of seeing the same FP “a lot of the time” [27]. Cultural access depended significantly on the patient’s age, a demographic data point which has already been found to be connected with evaluations of care [28,33]. The geographical access model highlighted as significant sex, retirement status, and self-assessed health status. Female patients perceived less access to health care than men. This result is very possibly linked to women’s restricted mobility caused by Slovenia’s poor public transit system, combined with the traditional arrangement of men being the primary, or even the only, driver in the household. Retirees also perceived geographical access to be worse than non-retirees. Retirees often experience transport as a challenging, or even insurmountable, obstacle to accessing PC. Self-perceived worse health status was associated with lower geographical access. This correlates to the fact that healthy patients require fewer and less frequent health-care services. Conversely, as found in an international study, patients who are less healthy raise the cost of health care and are more likely not to receive proper treatment [24]. The organizational access model exposed place of birth as significant: patients born outside Slovenia perceived the organizational access to be better than patients born in Slovenia. This is probably associated with the fact that there are many seasonal workers in Slovenia, mainly from former Yugoslavian countries. In the past two decades, governments in the countries of central and Eastern Europe have embarked on far-reaching reforms of financing, organisation, and delivery of health care [38]. Patients from abroad probably recognize that FP is the entrance point to the system [11,12]. In addition, this could also indicate that patients born in Slovenia are more demanding and critical users of PC services than those born outside Slovenia.

### Study limitations and strengths

Our study is subject to several limitations. The answers of patients who refused to be interviewed could not be included and their characteristics, health-seeking behaviour and perceptions of access may differ from survey respondents. Data from this study are cross-sectional, which does not allow for a demonstration of causality. Additionally, all explanatory variables were self-reported and unverified. Thus they are subject to recall and misclassification bias. The authors did not gather any of the data themselves, but had to rely on self-reported data. Limitations of self-reported data refer to patient self-assessment delivering several potential sources of bias, including cognitive limitations, selective memory, and exaggeration.

On the other hand, this study involved a large number of participants, making a strong case that the results can be generalised to the Slovenian population as a whole. In addition, there were only minor differences in the socio-demographic characteristics of the patients studied in comparison to the general population. Finally, this study included rural and urban general practitioner offices.

## Conclusions

The best access to PC is seen in the communicational and financial dimensions; the worst in the organizational. The financial access model showed no significant socio-demographic factors and it appears that patients in Slovenia have equal access to PC regardless of their financial background. In the organizational access model it was observed that patients born outside Slovenia perceived organizational access to be better than patients born in Slovenia. The problems detected can be the basis for improvement measures.

## Additional file

Additional file 1: Annex 1. Questions included in access to PC concept.
