# Supplementary material for: A cross-sectional study of socio-demographic factors associated with patient access to primary care in Slovenia
Source: Int J Equity Health. 2015 Apr 21;14:39. doi: 10.1186/s12939-015-0166-y (PMC4411768; doi:10.1186/s12939-015-0166-y)
Supplement: Additional file 1: Annex 1. — Questions included in access to PC concept. [file 12939_2015_166_MOESM1_ESM.docx]

**Annex 1:** Questions included in access to PC concept

| Access to PHC  dimension | PE question (code) | Reversed/recoded | Values |
| --- | --- | --- | --- |
| Communicational | *The doctor had my medical records at hand.* (medirec) | no/no | 1 = yes, 0 = no |
|  | *The doctor was polite.* (polite) | no/no | 1 = yes, 0 = no |
|  | *The doctor listened carefully to me.* (listen) | no/no | 1 = yes, 0 = no |
|  | *The doctor asked questions about my health problem.* (questhl) | no/no | 1 = yes, 0 = no |
|  | *The doctor took sufficient time.* (timesuf) | no/no | 1 = yes, 0 = no |
|  | *The doctor involved me in making decisions about treatment.* (involve) | no/no | 1 = yes, 0 = no |
| Cultural | *The doctor or staff showed disrespect because of your ethnic background.* (disrethn) | yes/no | 1 = no, 0 = yes |
|  | *The doctor or staff showed disrespect because of your gender.* (disrsex) | yes/no | 1 = no, 0 = yes |
|  | *The doctor or staff acted negatively to you.* (actneg) | yes/no | 1 = no, 0 = yes |
|  | *Other patients were treated better than you.* (bettertr) | yes/no | 1 = no, 0 = yes |
| Financial | *Postpone or abstain from a visit due to not having insurance.* (postpinsur) | yes/no | 1 = no, 0 = yes |
|  | *Postpone or abstain from a visit due to other financial reasons.* (postpfinan) | yes/no | 1 = no, 0 = yes |
| Geographical | *The practice is too far away from where I am living or working.* (farpract) | yes/no | 1 = no, 0 = yes |
|  | *How long does it usually take you to travel from your home to this practice?* (travel) | yes/no | 1 = less than 40 min, 0 = 40 min or more |
| Organizational | *If I need a home visit I can get one.* (homevis) | no/no | 1 = yes, 0 = no |
|  | *Was it easy to get the appointment?* (easyapp) | no/no | 1 = yes, 0 = no |
|  | *How many days did you wait for this visit?* (waitdays) | yes/yes | 1 = 7 days or less, 0 = more than a week |
|  | *Do you think it is too difficult to see a GP during evenings, nights and weekends?* (difoohr) | no/yes | 1 = no, 0 = yes |
